# Prisoners’ Educational Experiences in Five Different Prison Sports Programmes: A Research Note

**DOI:** 10.3390/ejihpe13100162

**Published:** 2023-10-23

**Authors:** Johannes Müller, Rosie Meek, Jonna Blessing, Michael Mutz

**Affiliations:** 1 Institute of Sport Science, Justus-Liebig-Universität Giessen, 35394 Giessen, Germany; jonna.blessing@sport.uni-giessen.de (J.B.); michael.mutz@sport.uni-giessen.de (M.M.); 2 Department of Law and Criminology, Royal Holloway University of London, Egham TW20 0EX, UK; r.meek@rhul.ac.uk

**Keywords:** incarceration, personal development, offender rehabilitation, social work, sports for development

## Abstract

Organized sports programmes offer manifold opportunities for learning and personal development. Prisoners in organized sports programmes could profit from these educational opportunities, which could eventually support their process of reintegration into society. However, research on the educational experiences of imprisoned individuals during organized sports activities is scarce. Using quantitative survey data (N = 568 adult male prisoners) collected within the scope of the Hessian Prison Sports Study in Hesse, Germany, the present study examines educational experiences that are instigated through participation in five different prison sports programmes (fitness, racket, and team sports, running groups, and strength training). The results show that participants reported few educational experiences. The most common experiences reported were learning to exert effort and acquiring health-related knowledge. The findings reveal distinct patterns for specific sports programmes. For instance, team sports more frequently address cooperation skills and fairness. This paper advocates for more attention to the educational potential of sports in prison settings, where sports outcomes should be better aligned with the desired educational outcomes.

## 1. Introduction

Researchers and educators often point to the educational value of organized sports programmes [1,2,3,4,5]. These programmes are assumed to facilitate knowledge acquisition, the practice of new social skills (e.g., cooperation, fairness), or the development of certain personality traits (e.g., self-efficacy). Systematic reviews show that sport can fulfill at least some of these educational and developmental potentials [6,7,8]. However, any of these outcomes may depend on a variety of influences and moderating factors, such as the type of sport, a supportive pedagogical arrangement, instructor qualification, etc., [1,6,9]. 

Prisoners in organized sports programmes may also profit from the educational potential of sports, which could in turn support their process of reintegration into society [10,11]. Scholars from sports pedagogy argue that those at risk of or engaging in offending could learn, for instance, emotion regulation, cooperation, perseverance, discipline, and the willingness to exert effort [12,13,14,15,16]. However, research on the educational experiences of prisoners during organized sports activities is scarce. The few studies that exist found that some specific sport interventions—from strength training to rugby or yoga classes—promote positive change in attitudes towards offending, help to reduce aggression levels and antisocial behavior, or lead to increased impulse control [17,18,19,20]. Furthermore, studies showed that sports programmes in prison can be a unique means to promote education [21] and contribute to learning rules and norms [22]. However, it is unlikely that prison sports programmes as different as strength training and yoga all yield similar effects, so it is recognized that comparative analyses of different programmes offer the potential to complement existing knowledge.

Morgan and colleagues [23] developed a theoretical model of sport-induced change processes that they apply to the prison context. They position certain educational experiences that foster individual development at the core of their model, all of which may be positively linked to an offender’s rehabilitation. Specifically, their model postulates that through participation in sports activities, offenders gain positive experiences such as a sense of achievement, a heightened ability to regulate emotions, or the feeling of doing something good. Moreover, sport provides space for learning and practicing and promotes commitment, dedication, and discipline. These experiences are then assumed to foster individual development in two ways. Firstly, pro-social qualities such as emotional control, resilience, and self-esteem are developed, which Morgan and colleagues [23] summarize with the concept of “positive psychological capital”. Secondly, sporting experiences can lead to the acquirement of new skills, motivation, and a recalibration of personal aspirations that facilitate the way into professional education and training programmes. Hence, sporting experiences could ideally also promote the development of human capital. The model states that the accumulation of both human and psychological capital is positively linked to desistance from crime. Sport is thus conceived as a suitable hook to initially engage offenders in an activity. After such an initial phase, a supportive climate created by coaches and trustworthy relationships in the sports activities are described as levers for further positive developmental experiences.

Although not intended to precisely test the proposed theory of change [23], the present study builds on this literature and explores some of the educational experiences that are key for the postulated change process. Hereby, we refer to those experiences made in organized sports programmes that potentially relate to educational outcomes, such as learning to regulate emotions, experiencing that effort pays off, or learning to take responsibility. Specifically, we examine the extent to which prisoners encounter different educational experiences during prison-organized sports activities. We further assess the extent to which these experiences vary between different prison sports programmes. Hence, this research aims to contribute to the state of knowledge by describing and analyzing prisoners’ educationally relevant experiences in a variety of different sports programmes.

## 2. Methods

### 2.1. Study Design and Data Collection

The Hessian Prison Sports Study (HePSS) provided the data used in this analysis [24]. The HePSS’s main objective was to gather empirical data on male prisoners’ sport, exercise, and health-related behaviors as well as on the athletic programmes offered in German prisons. For this aim, a cross-sectional quantitative survey was carried out between October 2021 and April 2022. The survey was conducted in all 12 prisons in Hesse, Germany, which overall house 2467 male inmates in regular custody (data from February 2022). Prisoners in preventive custody (after serving a sentence) and pre-trial detention were excluded. In smaller prisons (with <100 prisoners) all inmates were surveyed. In larger prisons, we randomly selected individual wards, in which all prisoners were then asked to participate in the study (cluster sampling). This strategy is justified because single wards usually contain a heterogeneous group of individuals. Prisoners with insufficient German language proficiency for reading and comprehending the questionnaire and consent form were excluded. All procedures received ethical approval from the local ethics committee of the Psychology and Sport Science Department of the Justus-Liebig-University Giessen, Germany.

We distributed questionnaires and consent forms to a total of 1672 prisoners using the sampling strategy described above. Participation in the study was entirely voluntary, and there were no benefits or drawbacks for the prisoners if they chose to participate or not. Prisoners had several days to fill out the questionnaire privately in their cells. Afterwards, they returned it in an envelope into a sealed box in order to maintain their anonymity. A total of 568 prisoners returned a completed questionnaire, representing a response rate of 34%, which is considered high for prisoner survey response rates [25].

The resulting sample includes inmates with an average age of 41 years (SD = 10.9, min = 22, max = 83). The incarceration periods, i.e., the periods from the beginning of the sentence to the time of the survey, ranged from <1 year (29%), up to 2 years (31%), up to 5 years (22%), to more than 5 years (19%). A total of 56% of respondents were serving their first sentence, whereas 44% were imprisoned for at least a second time.

### 2.2. Participation in Prison Sport Programmes

The questionnaire included a list of all organized sports programmes offered by the specific prison. This list of sports courses was prepared in a preliminary meeting with the responsible staff. For each sports course on the list, prisoners could then select whether or not they had participated in this course during their current prison sentence. In case the response was “yes”, they were then asked to report their participation frequency in the last few weeks from 1 = “no longer participate”, 2 = “only occasionally”, 3 = “often”, and 4 = “almost always”. We count those individuals as “regular participants” of a certain sports course, who stated a history of taking part as either “often” or “almost always”.

After collecting the data from 12 Hessian prisons, we assigned the sports programmes to five broader categories, namely fitness, racket, and team sports, running groups, and strength training. “Fitness sports” refers to staff-led sports activities in the prison gym that aim to improve general fitness, agility, and endurance, such as indoor cycling on stationary bikes, cardio circuit training, or high intensity interval training (HIIT, e.g., Tabata). “Racket sports” comprises mainly of two sports, table tennis and badminton. “Team sports” includes soccer, volleyball, basketball, and handball. Team sports are usually played at team strength with most of the time spent in free play. “Running groups” are organized running activities that take place in the outdoor areas within the prison, where often small running tracks exist. Whereas in some running groups inmates are given the opportunity to participate in guided running exercises, in others they run on their own. In the latter case, they usually decide for themselves how fast they run and do not necessarily run within groups. “Strength training” refers to all activities that take place in weight rooms, which are usually equipped with dumbbells, cable pull stations, dip stations, and pull-up bars. After a general instruction in weight training, detainees can use the weight room at set times under the supervision of prison staff.

### 2.3. Educational Experiences

We provided a list to prisoners with seven different experiences that are often considered to have educational value. This list included: (a) “Reflecting on rules and fair play”, (b) “Learning to take responsibility”, (c) “Learning to regulate emotions”, (d) “Learning about living healthy”, (e) “Cooperating with others in a group”, (f) “Experiencing that effort pays off”, and (g) “Mastering tasks I would not have thought possible”. Although this list was not developed as a tool to measure Morgan and colleagues’ [23] theory of change, the items are aligned with several components described in their work. For instance, emotion regulation, self-efficacy, and sense of achievement have a prominent role in their model. Their general reference to “learning” is specified in our list in relation to health and cooperation. Other educationally relevant experiences such as a reflection of fairness and rules extend the original model. Prisoners were asked to indicate on a 5-point Likert scale how accurate each item describes their experiences during prison sport activities (from 1 = “strongly disagree” to 5 = “strongly agree”).

### 2.4. Statistical Analysis

We first provide a descriptive overview of educational experiences based on prisoners’ reports. Then, ordinal regression models (polytomous universal model, PLUM, in IBM SPSS 28) are applied to test whether certain educational experiences correlate with specific sports programmes. Here, we estimate the effects by using strength training as the reference group. In these models, all variables that could be confounded with prisoners’ educational experiences in sports are included, such as age (in years), highest educational attainment (in five categories: 1 = “no degree”, 2 = “lower secondary school degree”, 3 = “medium secondary school degree”, 4 = “higher secondary school degree”, 5 = “tertiary/academic degree”), and the length of incarceration at the time of the survey (from 1 = “less than 3 months” to 7 = “>10 years”).

## 3. Results

### 3.1. Organized Sports Activities: Offers and Participation Rates

The number of different sports programmes per prison at the time of data collection varied between two and seven (M = 4.83; SD = 1.90). The differences are mainly related to the size of the prison and the personnel and equipment available. In some prisons, the number of sports activities was still limited at the time of data collection due to the COVID-19 pandemic. Most frequently, the prisons offer table tennis, fitness sports, and weight training (in 11 out of 12 prisons). Soccer (in seven prisons) and volleyball and badminton (in six prisons each) are also among the frequently offered activities. Running groups are available in 3 out of the 12 prisons.

Participation in organized sports activities is high overall, with 55% of prisoners stating that they regularly (“often” or “almost always”) participate in at least one of the prison’s sports programmes, whereas 45% of the inmates do not participate regularly in organized sports. Overall, 20% participate in one sport course, 18% in two sports courses, and 17% in three or more sports courses. The highest proportion of prisoners reported being involved in strength training (37%), followed by fitness sports (34%), racket sports (20%), and team sports (14%). Running groups reach a smaller proportion of prisoners (6%), although it should be noted that the survey took place in the autumn and winter months, when this activity is likely to be less popular.

### 3.2. Educational Experiences in Different Sports Activities

**Figure 1:**
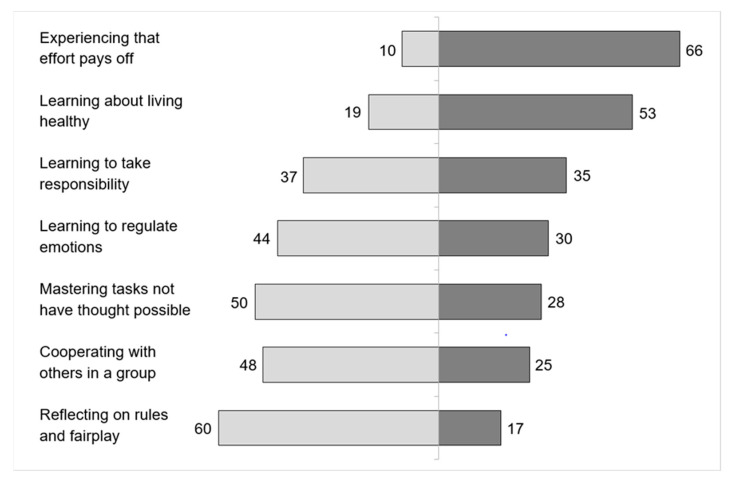
Educational experiences of prisoners in prison-organized sports activities. **Note**: Left side of the bar shows proportion of inmates (in %) who “strongly/somewhat disagree”; right side of the bar shows proportion who “strongly/somewhat agree”. The difference to 100% (of the sum of left and right bars) relates to prisoners who “neither agree nor disagree” with the respective statement.

Prisoners report educational experiences, but with clear distinctions depending on the experience in question (Figure 1). A proportion of 66% experienced that “effort pays off” and 53% report having learned about healthy lifestyles during sports programmes. These experiences are shared by the majority of those who report having participated in a sports program. Other educationally relevant experiences are only reported by a minority: 35% report having experienced a responsibility role during sports; 30% regard emotion regulation as a learning outcome of sports; 28% report mastery experiences; and 25% state that cooperation in a group is learned during prison sports. The lowest proportion (17%) agreed with the statement that a reflection of fairness and rules take place in sports programmes.

The PLUM models (Table 1) show that prisoners are more likely to report having certain educationally relevant experiences in sports when they participate in more prison-organized sports activities. Specifically, the models indicate significant associations between a prisoner’s self-reported engagement in sports courses and the chance to learn about a healthy lifestyle (b = 0.25), cooperate with others (b = 0.26), and experience that effort pays off (b = 0.31). Educational experiences also vary with the type of the sports program. Participation in fitness sports (in comparison with strength training) is associated with a significantly higher chance of reflecting on fair play (b = 0.55), learning about emotion regulation (b = 0.70), and learning about a healthy lifestyle (b = 0.66). In team sports, participants are more likely to reflect on fair play (b = 0.70), take responsibility (b = 0.48), improve emotion regulation (b = 0.58), and learn about health (b = 0.61). Most notably, however, a strong effect of team sports relates to prisoners’ self-reported ability to cooperate in a group (b = 1.52). The results for racket sports and running groups are not significant, with one exception in each case: participants in racket sports have a lower likelihood of reporting health-relevant learning outcomes (b = −0.94) and running group participants have a higher likelihood of reporting an ability to experience roles of responsibility (b = 1.34).

## 4. Discussion

Against the background of a lack of research on prison sports in the German penal system to date [26], the present analysis of the HePSS study explored educationally relevant experiences that prisoners report when participating in prison sports programmes. These experiences are presumably relevant for learning, personal development, and “psychological capital” and eventually may facilitate reintegration into society [23]. The key findings of this study show that half of the men in our sample reported participating regularly in at least one organized sports class. Although this is a relatively high proportion of regular participants, barriers to sporting activities do still exist, for instance, when smaller prisons offer only small range of activities or lack specific facilities for exercising. In this regard, our findings are compatible with studies that have identified barriers that impede prisoners’ participation in sporting activities [27].

Despite the high participation rate, not all prisoners report educationally relevant experiences. Whereas a substantial proportion has experienced that effort is worthwhile and acquired health-related knowledge, only a minority of prisoners report other educational experiences. Above all, cooperation in groups and reflections on rules and fair conduct seem to be recognized and reported less commonly. These findings are based on self-report data, so should be interpreted with caution. However, they do highlight a potential tension between prison policies and prison administrators’ assumption that sport fosters positive learning outcomes, such as acceptance of rules, self-control, or fair play [28], and the understanding of the educational potential of a sports initiative from the perspective of those prisoners who participate. This further highlights the need for clearly articulated theories of change for prison sports initiatives and the need for greater attention to effective evaluation methodologies.

The analyses also demonstrate that prisoners may have more educationally relevant experiences when they participate in more sports programmes, i.e., supporting the notion “the more, the better”. In addition, however, the educational experiences reported by prisoners varies with the type of sport. The results point to a potentially higher educational potential of team sports, perhaps because in this context prisoners are obliged to cooperate with each other, practice their emotion regulation, and observe rules. Fitness activities have a particular connection to health, although most activities offered tend to be high-intensity exercises (e.g., HIIT or indoor cycling). A particular strength of running groups appears to be that prisoners report taking more responsibility, presumably because in these groups they are less supervised and have more autonomy regarding the pace or whether they run alone or in a small group. However, this is a new area of focus with emerging research to build upon [29]. Overall, despite the fact that previous research has established that it is the relationships fostered through prison sports initiatives that have the greatest impact, our results also indicate that not all sports are necessarily the same. Typical experiences may depend on the sports activity prisoners participate in and this is something to take into account when designing (and articulating a theory of change for) a diverse range of prison sports initiatives.

This research has strengths and weaknesses: A particular strength is the comparatively large and representative sample, which is based on random selection procedures and includes 23% of the population of prisoners in regular custody in Hesse, Germany. The only known sample bias results from the fact that non-German speaking prisoners were excluded. However, we cannot rule out self-selection bias. Specifically, those prisoners motivated to participate in the research may have been those more oriented towards engaging with sport and thus have a vested interest in promoting the educational outcomes associated with such activities. The comparison between different organized sports offerings has also not been part of systematic analyses so far, although it proves relevant. As a major limitation, it must be noted that the HePSS did not collect information about “process variables”, such as the content and structure of sports lessons, the autonomy participants have for making their own decisions, the quality of instruction, the composition of the participants, or the social dynamics within the group. These variables, however, are likely to affect participant’s educational experiences. Another limitation is that the data are based on self-reports. Although prisoners stated that the questionnaire was comprehensible and they were alone when completing it, social desirability or memory lapses may still affect some responses. Finally, prisoners’ self-reported experiences should not be equated to the actual learning outcomes. Just as participants may overestimate learning effects in a sports activity, learning effects could also occur when participants do not even realize them in their self-perception, and thus they will not necessarily report them in studies such as these. Nevertheless, this study goes some way to meet the research need to develop a more nuanced understanding of the positive impact of prison sports initiatives in supporting educational and rehabilitative outcomes.

## 5. Conclusions

The present study shows that prison sports programmes reach many imprisoned men and are a popular activity during incarceration. It can be concluded from this finding that the sports programmes in Hessian prisons seem to meet the sports preferences of many prisoners. The relatively high participation rate is very positive, as only those inmates who participate in sport can potentially gain educational experiences. This also means that sport could act as a hook to engage men in meaningful activities that could promote not only education and rehabilitation but also healthy lifestyles.

With regard to the educational experiences in organized sport, however, not all prisoners report educationally relevant experiences. Most commonly, inmates report having learned that effort pays off and acquired health-related knowledge. Given the educational and rehabilitative potential often ascribed to sports, this finding is rather sobering and calls for critical reflections on the design and delivery of sports programmes. The link between sport provision and educational objectives is rarely explicitly stated or reflected upon in either research or policy. Similarly, there is little clarity about the pedagogical arrangements suitable for prison sports. Rather, a majority of those in charge seem to assume that sport will have a positive effect on prisoners quite incidentally and automatically. However, scholars urge us to “manage for outcomes”, that is, to intentionally design programmes to develop transferrable skills [30] (p. 110). This could include supplementing programmes with non-sport activities that open up further educational opportunities. Hence, fully realizing the educational potential of sports programmes requires that activities must be better targeted to the desired educational outcomes.

## Figures and Tables

**Table 1:** Prisoners’ educational experiences with different sports activities in prison. **Note**: Indicated are unstandardized regression coefficients (b). Models are based on N = 305 prisoners who regularly participate in at least one prison-organized sports program. Significance: + *p* < 0.10, * *p* < 0.05, ** *p* < 0.01, *** *p* < 0.001.

	Reflecting on Rules and Fairplay	Learning to Take Responsibility	Learning to Regulate Emotions	Learning about Living Healthy	Cooperating with Others in a Group	Experiencing That Effort Pays Off	Mastering Tasks Not Have Thought Possible
	*b* *(SE)*	*b* *(SE)*	*b* *(SE)*	*b* *(SE)*	*b* *(SE)*	*b* *(SE)*	*b* *(SE)*
**Sports participation**							
No. of regular sports activities in prison	0.06 (0.10)	0.05 (0.10)	0.08 (0.10)	0.25 * **(0.10)**	0.26 ** **(0.10)**	0.31 ** **(0.10)**	0.08 (0.10)
Fitness sports	0.55 * **(0.25)**	0.13 (0.25)	0.70 ** **(0.25)**	0.66 ** **(0.25)**	−0.04 (0.25)	0.35 (0.25)	0.01 (0.25)
Team sports	0.70 * **(0.28)**	**0.48** + **(0.27)**	0.58 * **(0.27)**	0.61 * **(0.28)**	1.52 *** **(0.29)**	−0.20 (0.28)	−0.07 (0.27)
Racket sports	0.31 (0.24)	0.30 (0.24)	−0.13 (0.24)	−0.94 *** (0.25)	0.25 (0.24)	−0.25 (0.24)	0.31 (0.24)
Running group	0.22 (0.41)	1.34 ** **(0.43)**	0.00 (0.41)	0.60 (0.43)	0.10 (0.41)	0.15 (0.43)	0.19 (0.41)
Strength training	*Ref.*	*Ref.*	*Ref.*	*Ref.*	*Ref.*	*Ref.*	*Ref.*
**Controls**							
Age	−0.00 (0.01)	0.00 (0.01)	−0.01 (0.01)	−0.01 (0.01)	0.03 ** **(0.01)**	−**0.02** + **(0.01)**	−0.01 (0.01)
Education	−0.08 (0.11)	−0.11 (0.11)	−0.09 (0.11)	−0.04 (0.11)	−**0.19** + **(0.11)**	0.00 (0.11)	−0.42 *** **(0.11)**
Previous length of incarceration	0.06 (0.08)	−0.09 (0.08)	−0.03 (0.08)	0.11 (0.08)	0.05 (0.08)	−0.06 (0.08)	0.06 (0.08)
**Pseudo-R²** **(Nagelkerke)**	**0.065**	**0.065**	**0.065**	**0.149**	**0.185**	**0.088**	**0.068**

## Data Availability

The data that support the findings of this study are available from the corresponding author upon reasonable request.
